# Significance and implications of accurate and proper citations in clinical research studies

**DOI:** 10.1016/j.amsu.2021.102841

**Published:** 2021-09-11

**Authors:** Micah Ngatuvai, Cody Autrey, Mark McKenny, Adel Elkbuli

**Affiliations:** Department of Surgery, Division of Trauma and Surgical Critical Care, Kendall Regional Medical Center, Miami, FL, USA; Department of Surgery, Division of Trauma and Surgical Critical Care, Kendall Regional Medical Center, Miami, FL, USA; Department of Surgery, University of South Florida, Tampa, FL, USA; Department of Surgery, Division of Trauma and Surgical Critical Care, Kendall Regional Medical Center, Miami, FL, USA

Citations are an essential component of clinical research studies. In health sciences most articles will refer to over 20 other peer-reviewed publications [1]. Citations are a core part of the entire research process. Citations fuel literature reviews [2,3] and they allow researchers to link their experiments to previous results and conclusions and establish credibility [2,4,5]. Citations can help authors contribute to the growing compilation of literature and prevent plagiarism [6,7]. However, prior studies have acknowledged a prevalence of improper citation [[8], [9], [10], [11]]. Studies report citation inaccuracy rates of approximately 20–26% in biomedical literature [8,9]. Some studies have also reported citation inaccuracies within field-specific journals such as pediatric orthopaedics [8], neurosurgery [12], spine surgery [13], and foot and ankle surgery [14]. This trend is especially alarming as citation inaccuracies can diminish research validity [15]. Recognizing the causes or instances of inaccurate citations can prevent further improper citation [16].

## 1 What are the root causes of citation inaccuracies?

Citation misuse can originate in literature reviews, as authors can find and choose references in arbitrary fashions. This can stem from keyword choice; many authors use broad keywords to garner a large audience, but this can hurt the number of references they receive [17]. Language can also be a barrier: one study found over 30% of systematic reviews excluded studies not published in English, which dramatically decreases the scope of literature reviews [18]. Researchers may also be biased towards articles with many citations. This can be described as the ‘Matthew effect:’ the more a paper is referenced, the more it will continue to be referenced [19,20]. This is concerning as citation rate of articles is not necessarily an indicator of quality or significance [21]. While papers with many citations continue to receive attention, less cited articles may be neglected, potentially hindering research advancements.

Literature reviews may also be influenced by other arbitrary factors. For example, an author's social media presence showed a positive correlation with the rate at which they were cited [22,23]. Another consideration for citation rate is the primacy effect, which describes how citations listed earlier in a study are used more frequently than those that appear toward the end of a study [24]. The reputation of the author(s), organization(s), journal, or country represented by a paper may also play a role in the number of times it is cited [25]. Some authors or groups of authors may receive an increased number of citations based on their production level or experience in the field of study [25]. Additionally, luck and last name may inevitably affect the rate at which someone is repeatedly cited.

## 2 What are examples of citation inaccuracies?

Selective citation, whether purposeful or subconscious, is an endemic problem [11,26,27]. Studies showing positive results are cited more often than those with neutral or negative results, a phenomenon known as citation bias [26]. This gives readers a biased view and overrepresents positive findings [26]. Other common forms of improper citation usage include: secondary citation, incorrect/opposite conclusion, back door invention, fact not found, and inaccurate population. Secondary citation, or “amplification,” is the act of citing a fact in a paper that was itself supported by a citation instead of going to the original article [16]. Amplification leads to the expansion of a belief without additional primary data [10]. Incorrect/opposite conclusion occurs when inaccurate or missing information is cited. Specifically, an author may cite an article presenting the opposite conclusion referred to in the study [16]. This error is especially detrimental as studies make claims contradicting the citation, yet this contradiction may be further perpetuated in future studies via amplification. “Back door invention” is the error of citing abstracts while leading the reader to believe it is a peer-reviewed article [10]. Fact not found consists of a claim that a cited article has stated a fact or statistic, when in reality did not mention it at all and is therefore unsupported [16]. Lastly, inaccurate population involves the referencing of a study which may have found the results reported, but the results obtained in the cited article may not be generalizable to the population in the new study [16]. Research has also shown that citation of retracted studies occurs in many fields and these studies are often cited positively [[28], [29], [30], [31]]. One study showed that even after 5 years, retracted studies by an author were still being cited, with only 25% of citations acknowledging it had been retracted [31].

## 3 What can be done to prevent or correct citation inaccuracies?

Solutions to many of these problems have been shared [[15], [16], [17],^22^,^23^]. Authors can help others find their research by using targeted rather than generic keywords [17]. Similarly, a social media presence by journals and authors may be warranted to improve discoverability [22,23]. While not feasible for all literature reviews, machine learning has been used in finding relevant references for systematic reviews [32]. In short, machine learning is a form of artificial intelligence that allows systems to create algorithms based on data received. Future improvements in machine learning may allow for widespread use in finding and citing references in a way that is both efficient and accurate. One solution that has been proposed is the development of a tool named MyCites [33]. This tool would allow for the ability to mark citations as inaccurate and have these notations travel with the digital document so that future readers are aware of the accuracy of any contested citations [33]. These capabilities may help to stifle early citation inaccuracies and prevent the amplification of inaccurate citations.

Given the prevalence of citation inaccuracies, it is imperative those involved in the peer review process review submissions with an eye toward citation accuracy. At the start, authors must thoroughly recheck their citations and verify the relevance and validity of each reference. [34] One survey showed that only 4% of published scientists regularly check citations in articles they read [3]. Some authors have suggested that a simple checklist would avoid most errors [16]. It has also been suggested that editors develop training courses for authors outlining the acceptable citation styles pertinent to a particular journal [34]. Reviewers are in the unique role of making sure that new submissions are, evidence-based, in publishable condition, and add to the current body of knowledge. Such responsibility also includes evaluating the references of these submissions and suggesting the alteration, removal, or addition of references which would ensure citation accuracy [34]. The peer review process can help correct citation mistakes, especially through increased spot checks by editors/reviewers [9]. Lastly, the publisher's role in citation accuracy has started to include the use of software to process, link, and check the quality of references. [35] The increased utilization of new technology to verify citation accuracy will be of great benefit to both researchers and readers.

We also seek to share common guidelines for proper citation. First, ensure the citation provides correct publication details, including name, article title, and journal [15]. Second, the citation must substantiate the claim [15]. Next, authors should use unbiased sources that provide reliable data [15]. Articles from prestigious journals should not be assumed as reliable; analysis of the article itself is critical [15]. Additionally, be mindful of reconciling evidence. Authors should present the information in an objective manner [15]. In research it is crucial for “evidence to guide conclusions.” [36] Citations are an important part of the scientific process. They allow researchers to support and share findings, helping to further innovation. However, citations can be misused, slowing progress in clinical research and circulating unsupported beliefs. Many problems with citations can be fixed with increased attention to detail by authors and editors, ultimately strengthening credibility of the literature.

## Ethical approval

Not applicable.

## Sources of funding

None.

## Author contribution

Study design and conception: AE. Data collection, interpretation and analysis: MN, CA, AE. Manuscript preparation: MN, CA, AE, MM. Critical revision of manuscript: MN, CA, MM, AE. All authors read and approved the final manuscript.

## Trial registry number

1. Name of the registry:
2. Unique Identifying number or registration ID:
3. Hyperlink to the registration (must be publicly accessible):

Not applicable.

## Guarantor

Adel Elkbuli.

Mark McKenney

## Declaration of competing interest

None.
